# A Computed Tomographic and Pathological Study of Equine Cheek Teeth Infundibulae Extracted From Asymptomatic Horses. Part 2: MicroCT, Gross, and Histological Findings

**DOI:** 10.3389/fvets.2019.00125

**Published:** 2019-04-26

**Authors:** Apryle Horbal, Sionagh Smith, Padraic M. Dixon

**Affiliations:** The Royal (Dick) School of Veterinary Studies and The Roslin Institute, The University of Edinburgh, Midlothian, United Kingdom

**Keywords:** equine dentistry, equine dental imaging, equine infundibular pathology, equine dental pathology, equine dental caries

## Abstract

**Background:** Equine maxillary cheek teeth infundibulae are frequently affected by developmental and acquired disorders, but the imaging, gross, and histological features of normal and abnormal infundibulae remain incompletely understood.

**Objective:** To perform MicroCT, gross examination, and histology on sectioned teeth and compare the imaging and anatomical findings.

**Study design:** *Ex vivo* original study.

**Methods:** Eight maxillary cheek teeth of different ages and with varying grades of unilateral (*n* = 5) or bilateral (*n* = 3) occlusal infundibular caries were extracted from equine heads obtained from an abattoir. The teeth were imaged by MicroCT, then transversely sectioned and grossly and histologically examined, with the imaging and gross and histological anatomical findings examined and compared.

**Results:** Fifteen infundibulae, including two without occlusal caries had subocclusal cemental hypoplasia or caries. One infundibulum without occlusal caries had no subocclusal cemental defects. Histologically, hypoplastic cemental areas consisted of irregularly-shaped, wide central channels, with multiple, large, cylindrical side-branches that extended peripherally to a variable extent. Cementum with extensive, wide, empty channels, and cementum with a more irregular moth-eaten appearance had dark or eroded gross appearance and a low HU on CT. Some infundibulae had cement-free areas that only contained fragments of collagen-like material, especially at their apical aspects (apical cemental hypoplasia). Carious subocclusal areas had connections with the occlusal surface and had disrupted cemental architecture, including of their central vascular channel that, along with their side branches, contained degraded food, and cemental debris.

**Main Limitations:** No clinical histories or accurate ages were available for these eight teeth.

**Conclusions:** Hypoplastic cemental lesions, including at central linear and apical sites, histologically contain areas with multiple wide-branched, cylindrical channels or even areas of total cement hypoplasia visible on gross sections When such cemental defects contact the occlusal surface due to normal wear, food impaction, and caries can ensue.

## Introduction

Anatomical (1, 2) and computed tomographic (CT) studies (3–6) have shown that up to 90% of equine cheek teeth infundibulae, in particular the rostral (mesial) infundibulae of the Triadan 09 position, are incompletely filled with normal cementum, that preferably should completely fill the infundibulum. These infundibular defects, initially developmental in origin, include the very common presence of a fine central cemental defect (variously termed “central vascular channel,” “vascular channel,” or “central linear defect”) or larger areas of discolored hypoplastic cementum or even a total absence of cementum, as described (1, 2, 7) and also reported in the companion article (8).

Caries of infundibular cementum is an acquired, bacterial-acid mediated disorder that appears to preferentially affect infundibulae already affected by developmental defects that later trap food (8–10). Infundibular cemental caries can extend to the adjacent infundibular enamel and even to dentine and pulp where it can cause apical infection (without fracture development) (2, 7, 9–14). Maxillary cheek teeth can develop midline sagittal fractures that were initially termed “idiopathic cheek teeth fractures” (15) but later renamed *caries-related infundibular fractures* (12).

Dacre et al. (11) and later Suske et al. (3, 7) have described the gross and histological findings in maxillary cheek teeth infundibulae with various developmental and acquired cemental defects that predisposed to the above serious clinical disorders. Despite these studies, our knowledge of equine cheek teeth infundibulae in health and disease remains incomplete. The aim of this study was to examine the microCT characteristics of cheek teeth infundibulae with and without occlusal infundibular caries in horses of different ages and also to compare the gross, microCT and histological appearances of a range of infundibular appearances, to allow a more objective assessment of infundibular CT imaging.

## Materials and Methods

### Micro Computed Tomographic (MicroCT) Imaging

The eight cheek teeth used in this study were from equine heads with unknown histories obtained from an abattoir and included Triadan 08 (*n* = 1), 09 (*n* = 6), 10 (*n* = 1) from age groups <5 years (*n* = 2); 5–10 years (*n* = 2); 10–15 years (*n* = 1); 15–20 years (*n* = 2); >20 years (*n* = 1). Occlusal caries was present in 13 of the 16 infundibulae of these teeth, as follows: grade 1 (*n* = 10), grade 2 (*n* = 1), and grade 3 (*n* = 2) caries. Micro computed tomographic (MicroCT) scanning (XTreme CT, Scanco Medical AG, Bruttisellen, Switzerland) was performed at an isotropic spatial resolution of 82 μm. Acquired images were examined using OsiriX^©^ software. The attenuation of normal appearing infundibular cementum and of infundibular cemental lesions was measured in Hounsfield Units (HU) utilizing an ROI measurement tool on sagittal, two-dimensional images using OsiriX^©^ software.

### Gross Examination and Histological Preparation

Transverse sections of teeth, circa 5 mm thick were cut from sites selected on MicroCT images and were visually examined and photographed as described (14). Areas of hypointense or otherwise irregular cementum detected on MicroCT imaging and gross visual examination were later examined by low power microscopy allowing areas of normal and abnormal cementum to be selected for high-power microscopy and imaging. Sections were then decalcified and prepared for histology with H& E staining as described (11, 14).

## Results

### MicroCT Findings

Fifteen of the 16 infundibulae, including two of three without occlusal caries had subocclusal cemental lesions identified on MicroCT, with only one infundibulum being completely filled with normal appearing cementum. The eight rostral (mesial) infundibulae had cemental defects including central linear defects in 7/8, that was combined with apical cemental hypoplasia in 5/7 and caries in 2/7. One rostral infundibulum had marked caries with no remaining cement. The eight caudal (distal) infundibulae had apical cemental hypoplasia in 1/8; central linear defects in 4/8 with concurrent apical cemental hypoplasia in two; caries in two and one infundibulum had no lesions.

### Gross Visual Examination and Histological Findings

**Figure 1:**
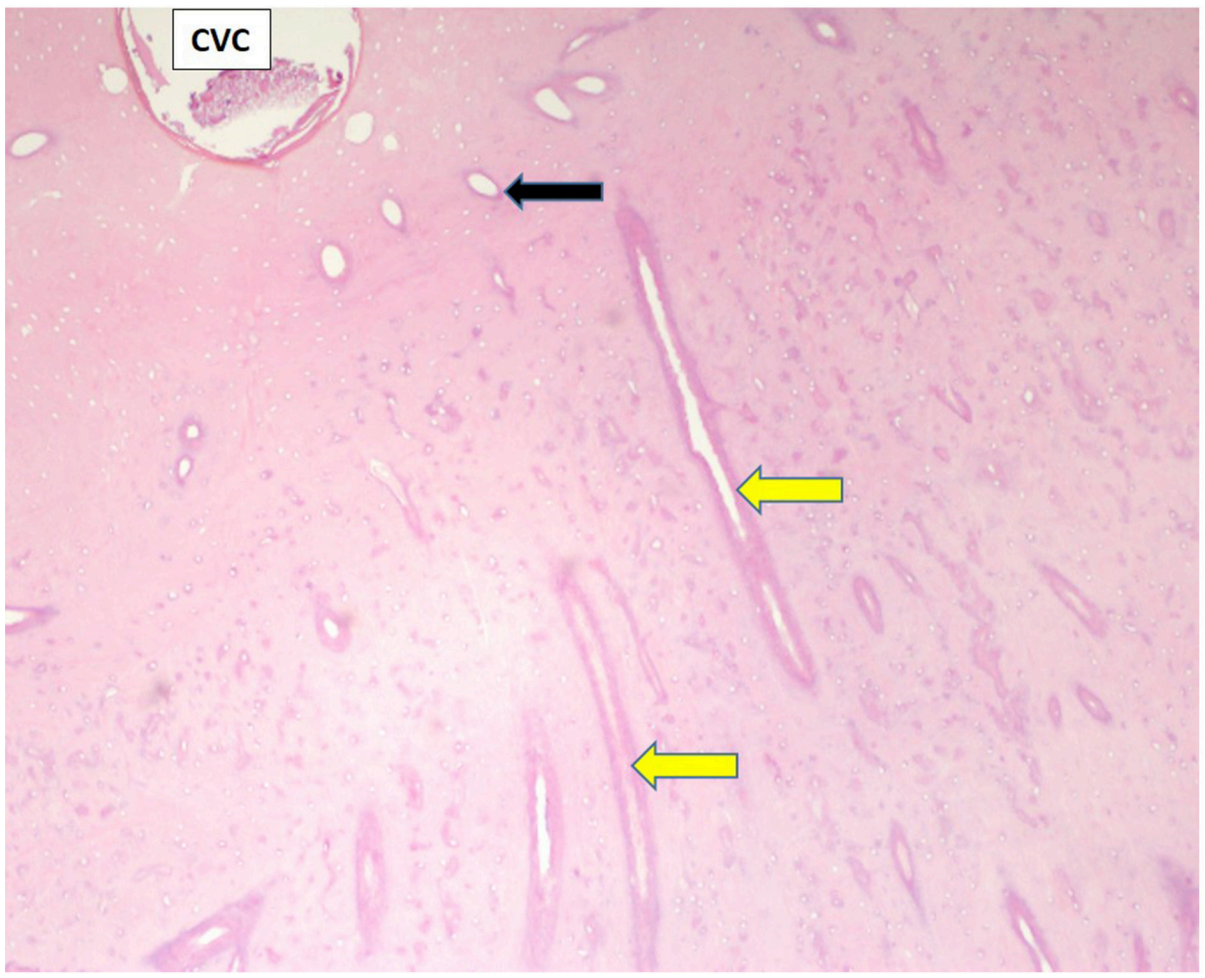
Transverse histological section of part of a grossly normal infundibulum. A small, round central vascular channel (CVC) at top left of image contains small amounts of degraded cementum. This infundibulum is filled with relatively dense cementum containing empty, relatively small, mainly transversely oriented lateral branches from the CVC (yellow arrows) but also some more vertically, and obliquely oriented small lateral branches (black arrow). (H&E; x 40 magnification when image is 140 mm wide).

**Figure 2:**
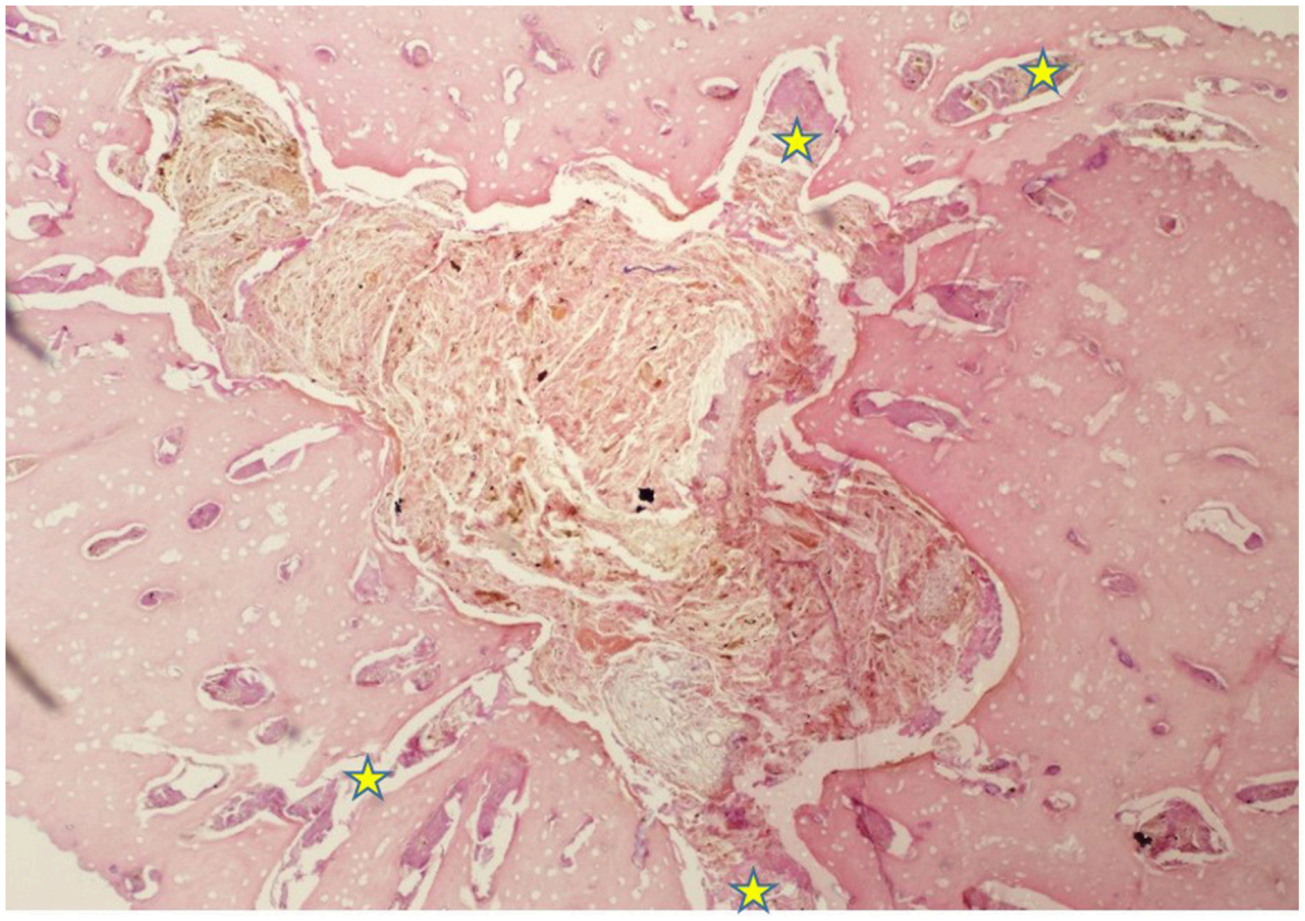
H&E stained transverse section of a central, longitudinally-oriented, subocclusal cemental defect of a rostral infundibulum that had occlusal caries. The image highlights a triangular cemental defect (central vascular channel) tightly packed with plant material, and surrounded by histologically normal cementum, with well-defined margins, indicating that this was a developmental rather than carious cemental defect. Some food material and minimal amounts of cellular debris and cemental fragments are also present in many smoothly-outlined, horizontally oriented lateral channels (yellow stars) branching off the central defect. Under the appropriate conditions, this deeper area of the infundibulum would later have developed caries (H&E; x 40 magnification when image is 140 mm wide).

A transverse histological section of a grossly normal infundibulum with a “normal” central vascular channel from a control tooth (no infundibular caries or subocclusal cemental defect) is shown in Figure 1. An example of a histological image of a food debris-filled, central linear cemental defect (linear defect wider than a “normal” central vascular channel) as described in an accompanying article (8) but without surrounding cemental caries is shown in Figure 2.

**Figure 3:**
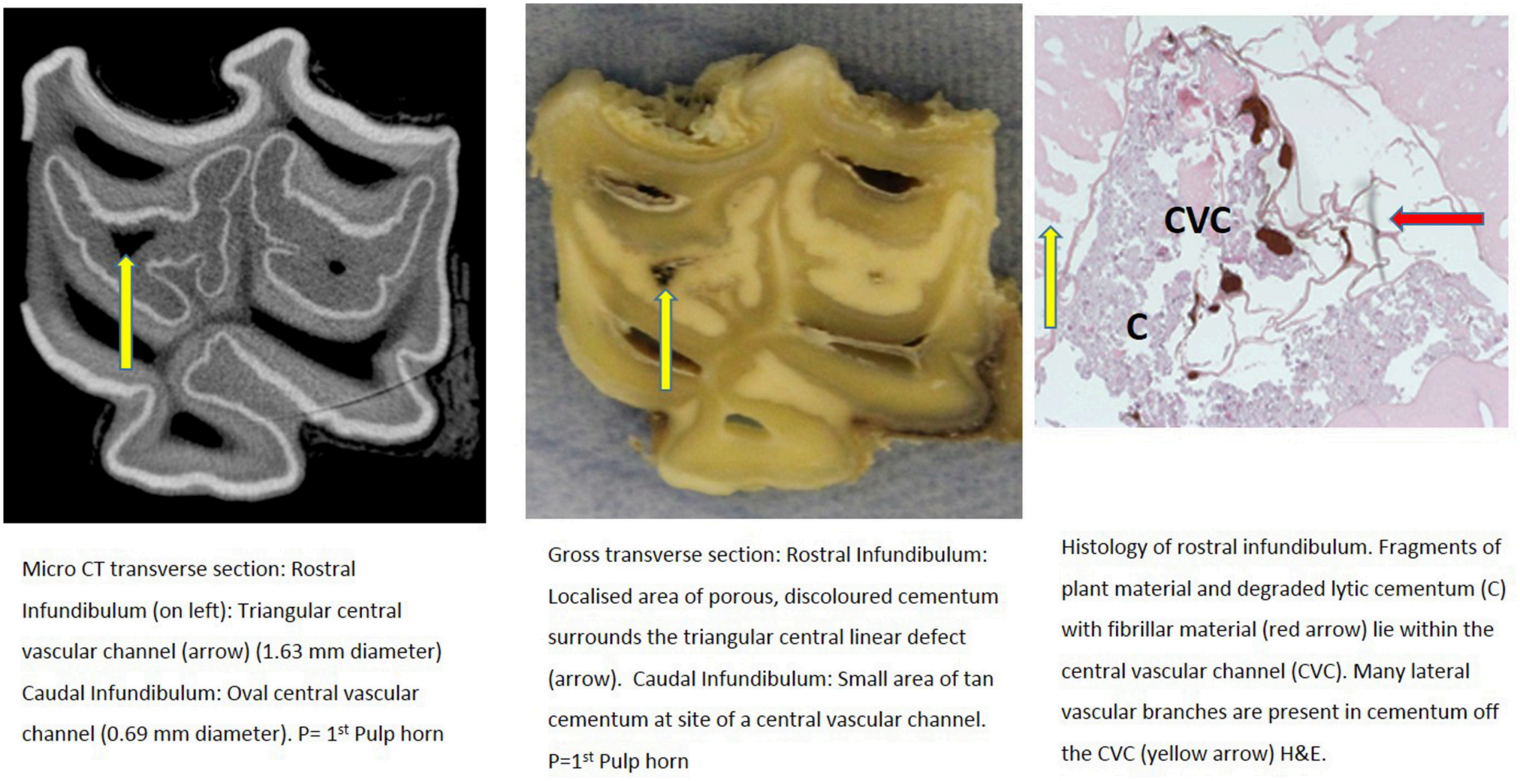
Tooth A, 30 mm subocclusally. Transverse microCT image and gross section (both x2 magnification—editor when this image is 19.6 cm wide), and histological section (H&E, x10 magnification). The rostral (mesial) infundibulum lies on the left of the microCT and gross section images.

**Figure 4:**
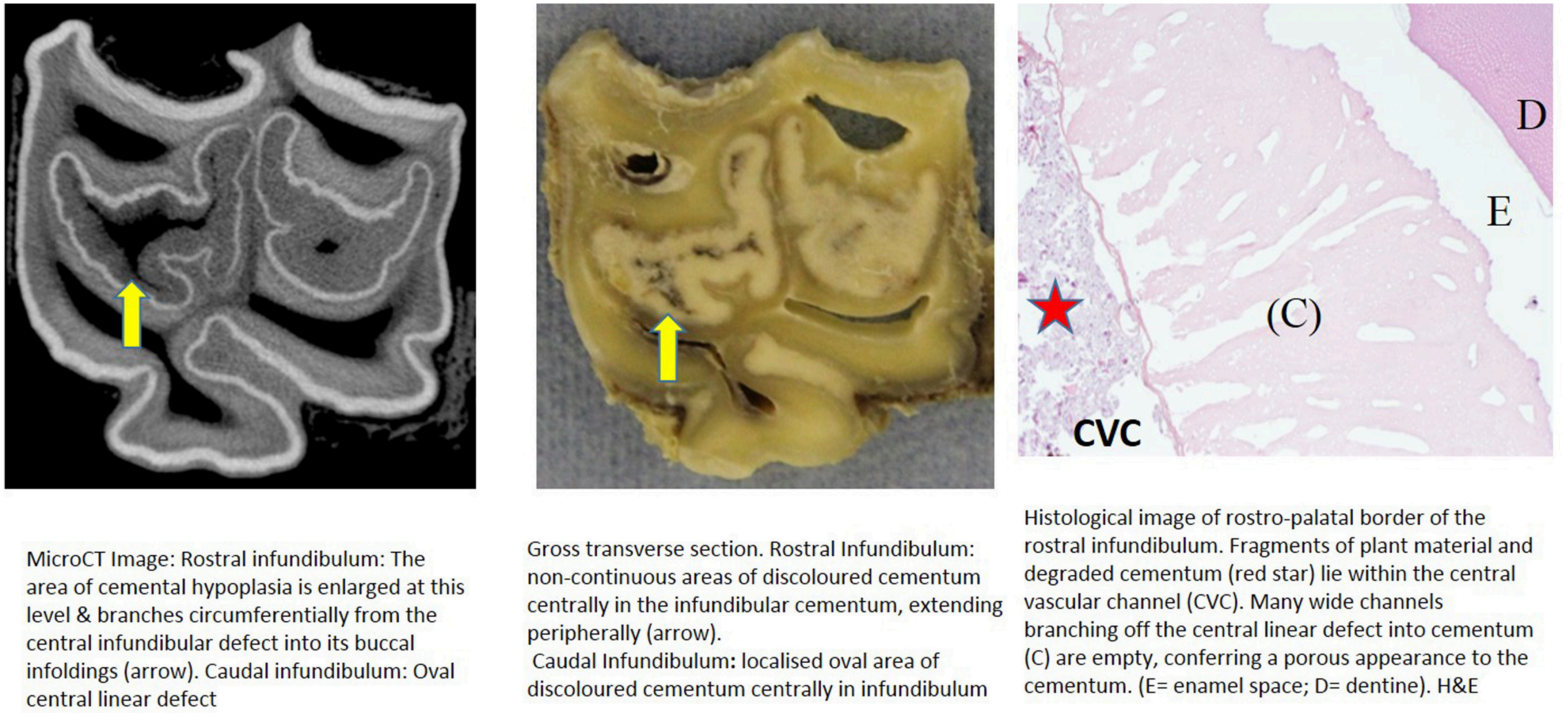
Tooth A, 40 mm subocclusally. Transverse microCT image and gross section (both x2 magnification—editor when image is 19.6 cm wide) and histological section (H&E, x 10 magnification). The rostral (mesial) infundibulum lies on the left of the microCT and gross section images.

**Figure 5:**
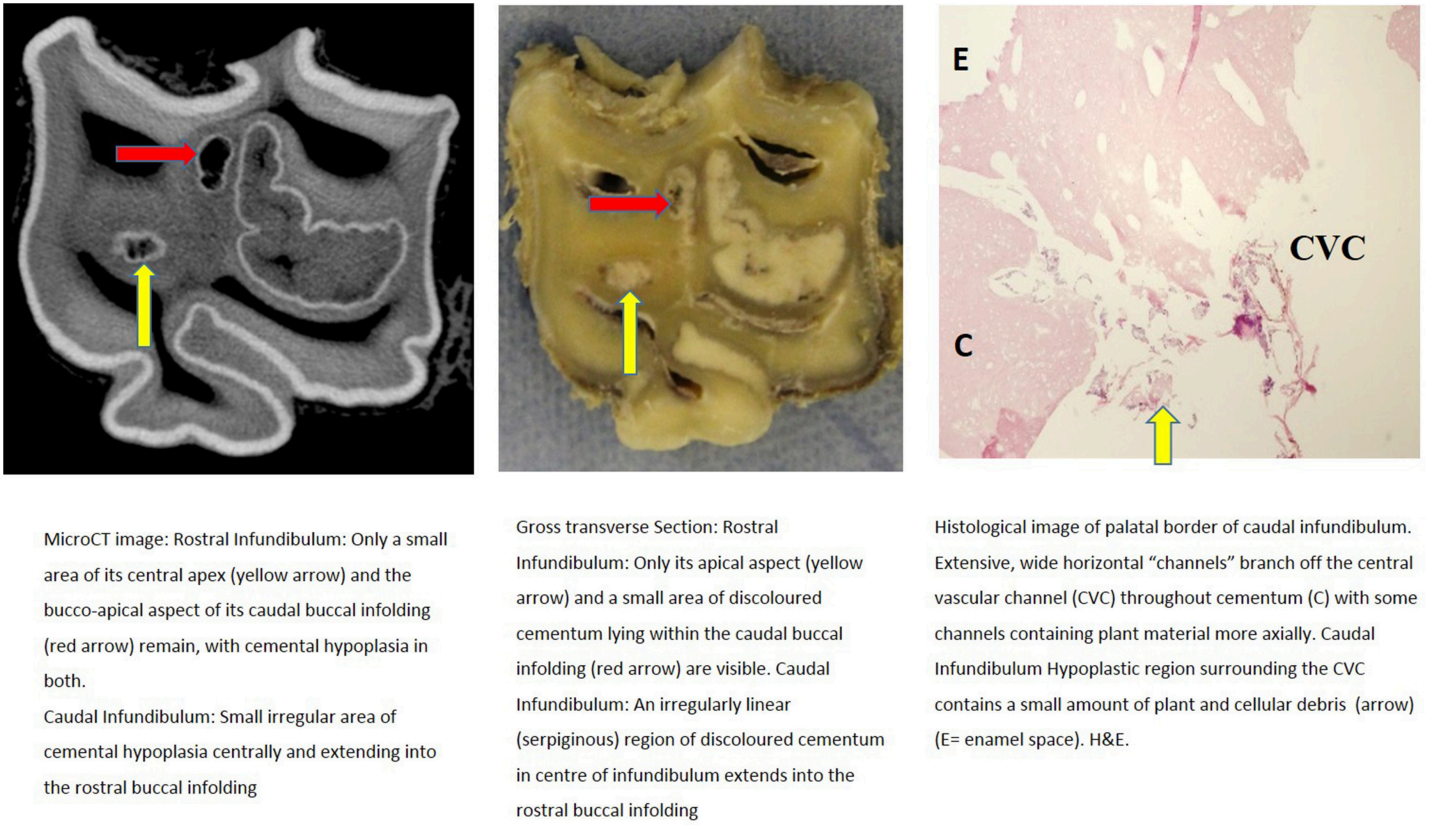
Tooth A, 55 mm subocclusally. Transverse microCT image and gross section (both x2 magnification—editor when image is 19.6 cm wide) and histological section (H&E, x 10 magnification). The rostral (mesial) infundibulum lies on the left of the microCT and gross section images.

**Figure 6:**
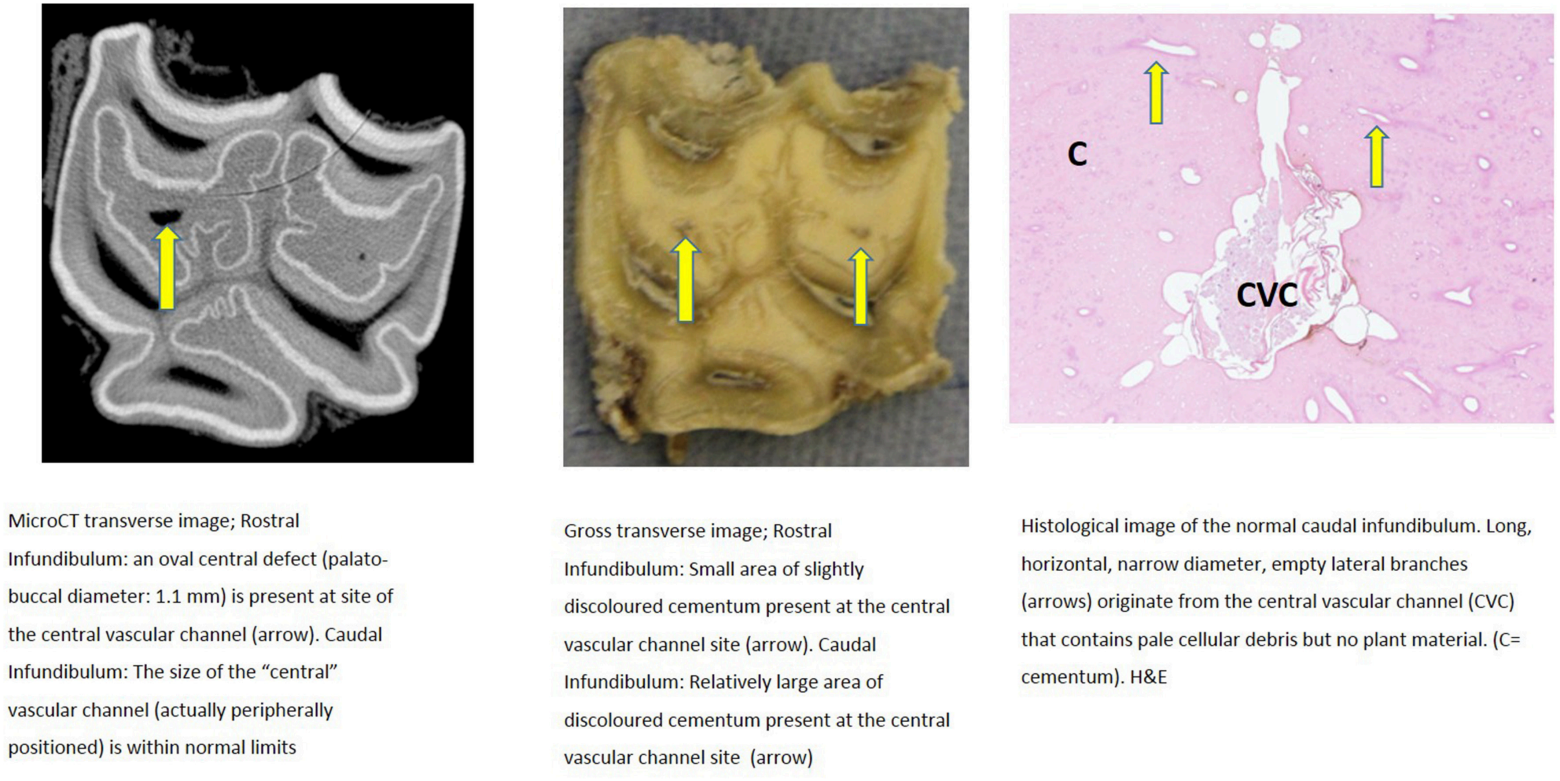
Tooth B, 20 mm subocclusally. Transverse microCT image and gross section (both x2 magnification—editor when image is 19.6 cm wide) and histological section (H&E, x 9 magnification). The rostral (mesial) infundibulum lies on the left of the microCT and gross section images.

**Figure 7:**
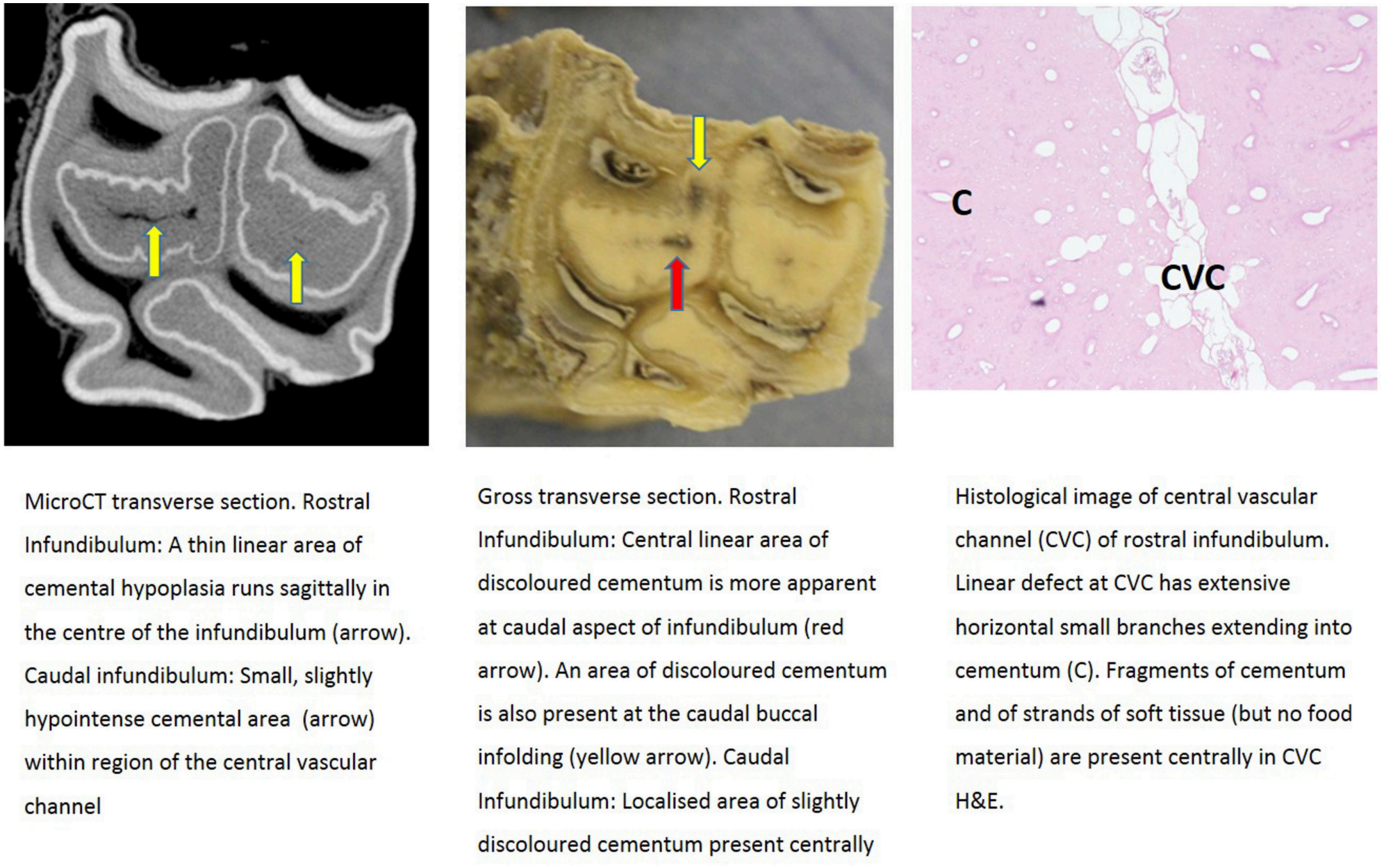
Tooth B, 50 mm subocclusally. Transverse microCT image and gross section (both x2 magnification—editor when image is 19.6 cm wide) and histological section (H&E, x 10 magnification). The rostral (mesial) infundibulum lies on the left of the microCT and gross section images.

**Figure 8:**
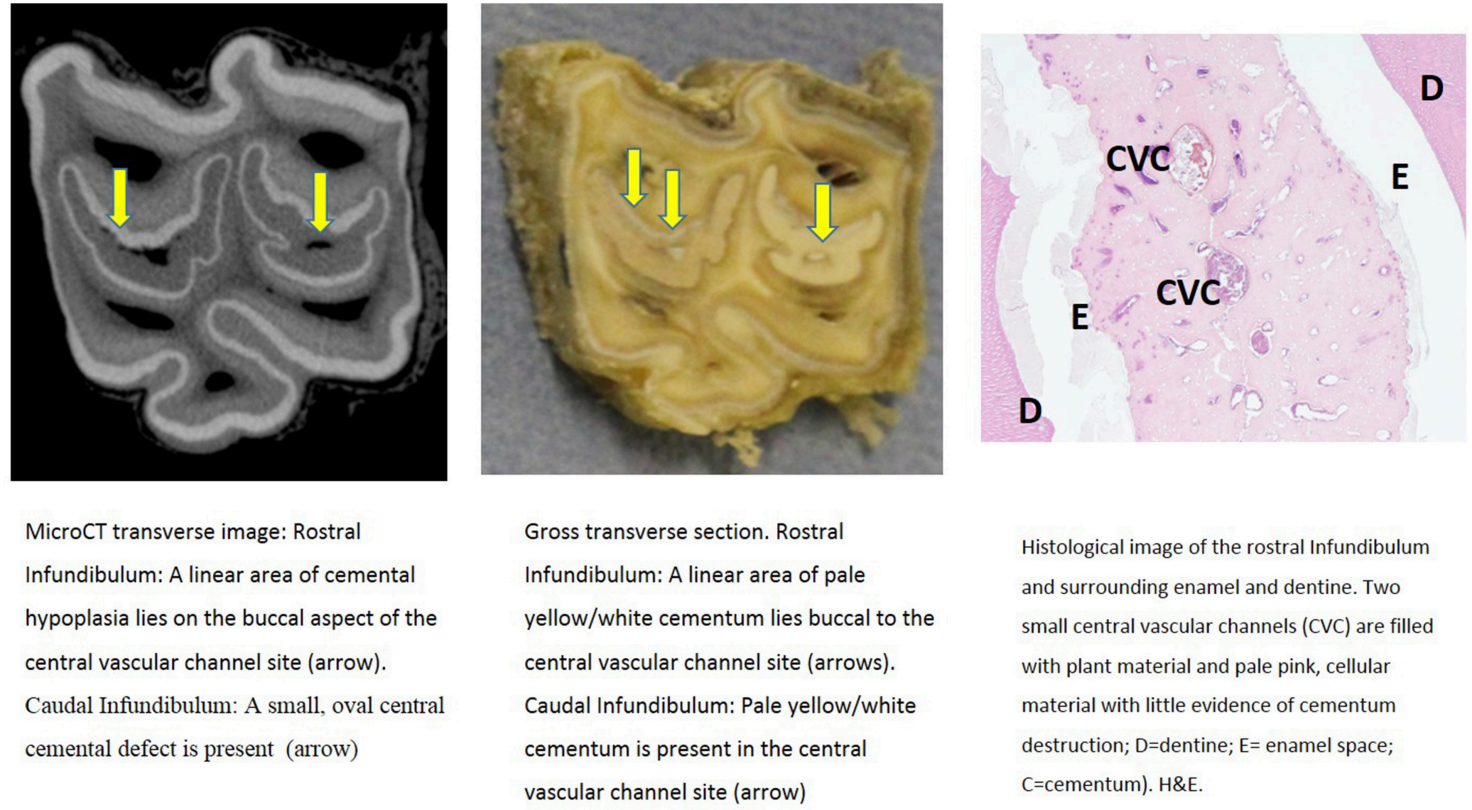
Tooth C, 15 mm subocclusally. Transverse microCT image and gross section (both x2 magnification—editor when image is 19.6 cm wide) and histological section (H&E, x 4.5 magnification). The rostral (mesial) infundibulum lies on the left of the microCT and gross section images.

**Figure 9:**
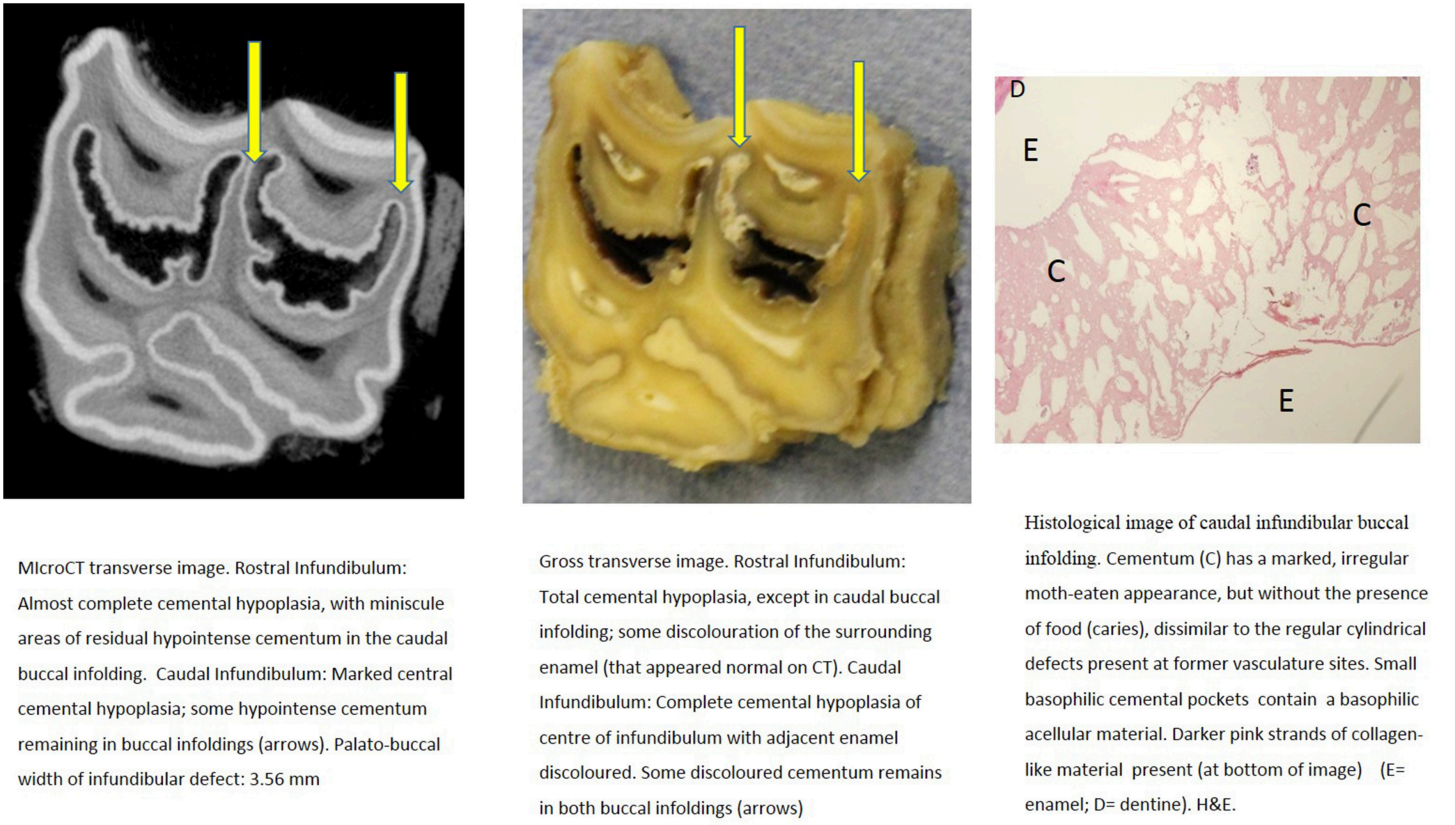
Tooth C, 30 mm subocclusally. Transverse microCT image and gross section (both x2 magnification—editor when image is 19.6 cm wide) and histological section (H&E, x 10 magnification). The rostral (mesial) infundibulum lies on the left of the microCT and gross section images.

**Figure 10:**
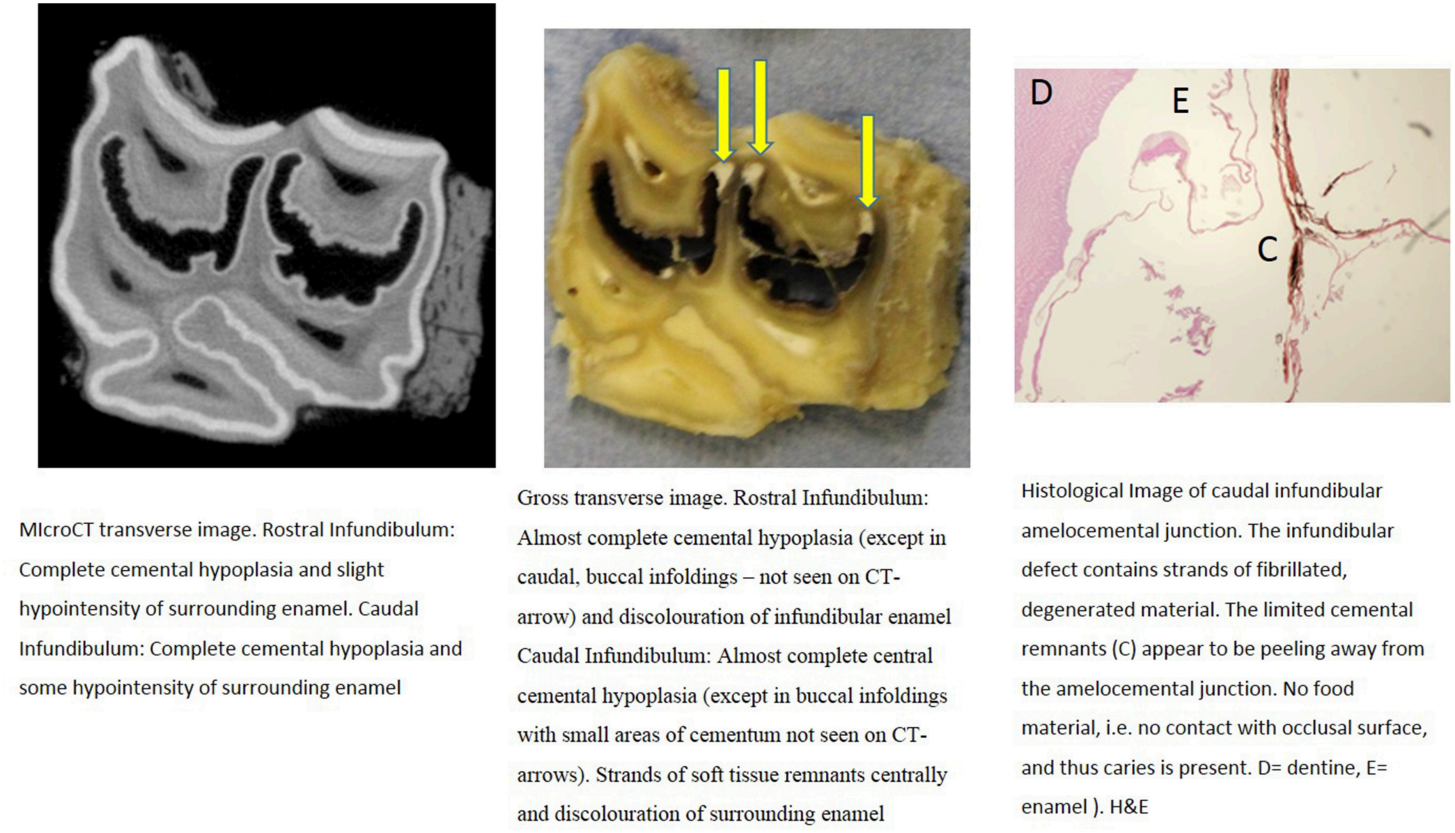
Tooth D, 30 mm subocclusally. Transverse microCT image and gross section (both x2 magnification—editor when image is 19.6 cm wide) and histological section (H&E, x 10 magnification). The rostral (mesial) infundibulum lies on the left of the microCT and gross section images.

**Figure 11:**
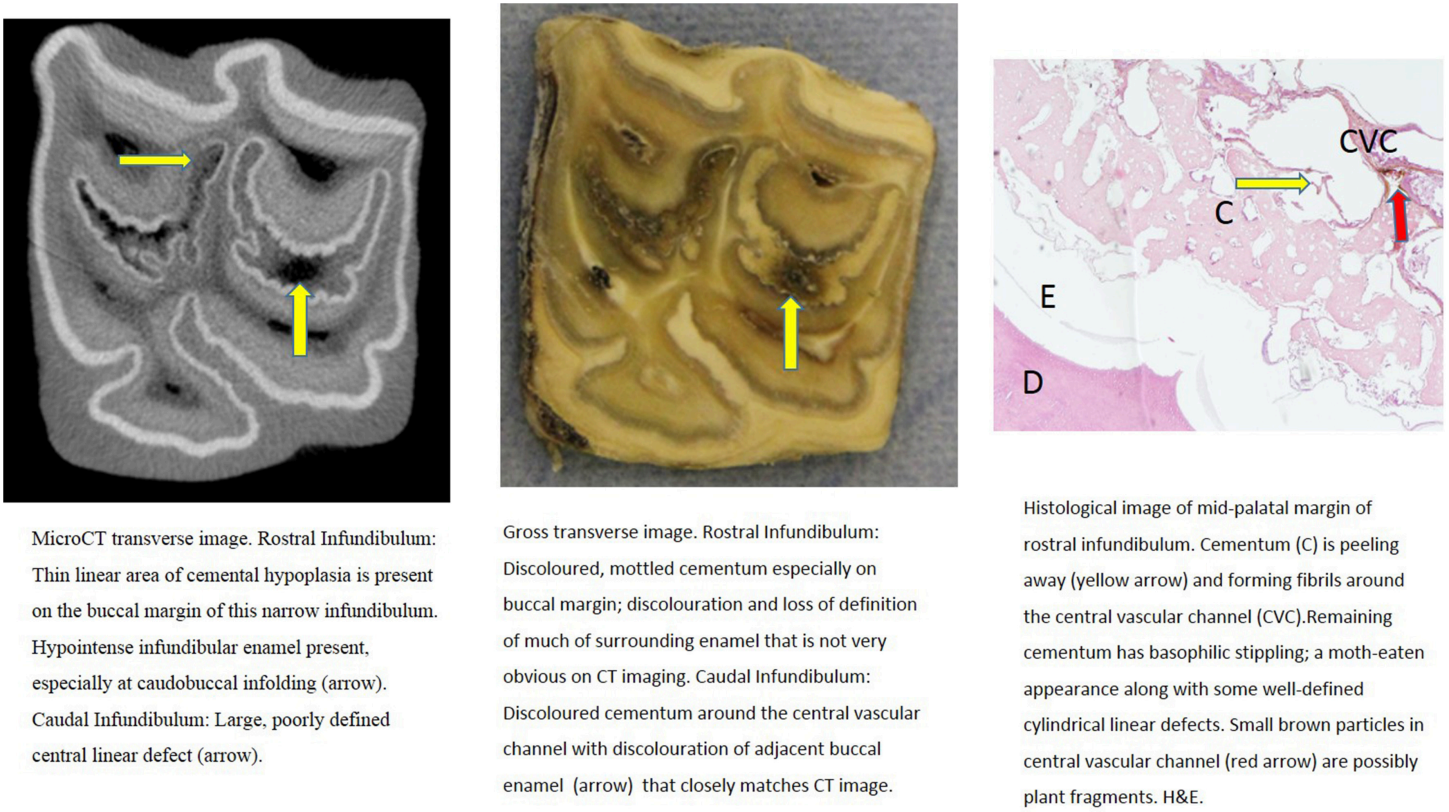
Tooth E, 15 mm subocclusally. Transverse microCT image and gross section (both x2 magnification—editor when image is 19.6 cm wide) and histological section (H&E, x 10 magnification). The rostral (mesial) infundibulum lies on the left of the microCT and gross section images.

Further descriptions of infundibular histology of maxillary cheek teeth with infundibular cemental hypoplasia and/or caries and corresponding MicroCT and gross images are presented in Figures 3–**11**. These panels of figures include MicroCT, gross, and histological images of the same teeth at different subocclusal levels for example in: Figure 3—(Tooth A; 30 mm subocclusally) and Figure 4 (tooth A; 40 mm subocclusally) and **Figure 8** (tooth C; 15 mm subocclusally) and **Figure 9** (Tooth C; 30 mm subocclusally). Comparison of images of these teeth at different levels shows the expansion of linear cemental lesions (likely in areas of developmental hypoplasia) more apically.

Subocclusal sections that appeared to have areas of almost total cemental hypoplasia on microCT with marked hypointensity had corresponding grossly recognizable defective or absent cementum at these same sites (e.g., Figures 3, **9**, **10**). However, other microCT images showed areas of marked cemental hypointensity that were larger than the lesions observed on gross and histological sections (Figures 4, **7**, **8**).

Gross examination of these 16 transverse sections showed the infundibulae to be variably filled with cementum ranging from dense, cream-colored normal cementum with just a fine central vascular channel; to tan, brown, or even black, variably porous cementum; to areas with partial (Figures 3–**8**, **11**) or total absence of cementum, especially apically, with just fragments of connective-type tissue present in the infundibulum (**Figures 9**, **10**).

Histological examinations showed that normal cementum had limited numbers of small side channels branching off the central vascular channel (Figures 1, **6**–**8**). In contrast, areas of hypointense cementum contained higher numbers of much wider channels branching off the consistently present “central” vascular channel that was not always centrally positioned (Figures 2–5, **11**). These lateral channels appeared to be the sites of former lateral branches of the large central blood vessel that supported cementum deposition more peripherally that were not replaced with cementum during development. Some such areas had a dense, smooth bordered central vascular channel, and side branches which, even though they often contained food material, had minimal evidence of carious destruction (Figure 2). However, if the horses had lived longer, it is likely that bacterial degradation of this cementum, i.e., caries would have occurred. Larger areas completely devoid of cementum (or plant material) were recognized both apically and centrally, sometimes containing fibrillar, collagenous-type material, possibly remnants of the enamel organ, and/or the dental follicle tissue (3) (e.g., Figures 5, **10**, **11**) indicative of developmental cemental hypoplastic defects.

Some areas of porous cementum, even deep within the infundibulum (Figure 2) without obvious caries, were filled with plant material that likely was the cause of much of the discolored carious cementum, but some hypoplastic areas without any occlusal connection (and thus not containing detectable food) also had strands of a basophilic collagenous-type material (Figures 6, 7). The grossly apparent, dark staining in these areas (Figures 6, 7) did not appear to be of bacterial or food origin, and was possibly due to residual porphyrin from the previous blood supply (7). Carious areas contained food material with disintegrating cementum in which there was loss of a well-defined central vascular channel and side branches (e.g., Figure 4). The most carious affected tooth (Tooth F), disintegrated during decalcification and could not be histologically examined.

### MicroCT Assessment of Infundibular Contents Density

The density of rostral and caudal infundibular cementum that appeared normal on gross and CT imaging was obtained from six teeth at different subocclusal levels. Cemental density could not be assessed in Tooth D (**Figure 10**) that had gross cemental hypoplasia/aplasia or in a Tooth F with marked caries and no cementum.

Normal appearing cementum (*n* = 17 sites) had a median density of 3,438 Hounsfield units (HU), (range: 2,029–3,725 HU; total range of 1,696 HU). Areas of normal appearing cementum, including an area with a cemental HU values of 3,578 (caudal infundibulum of tooth A, 30 mm subocclusally—Figure 3) and an area with a cemental HU value of 3,529 (caudal infundibulum of tooth B, 50 mm subocclusally—Figure 7) were histologically noted to have small, well-defined central vascular channels and side branches such as shown in Figures 1, 6–8. More occlusal cementum in six infundibulae had a median HU value of 3,601, while 22 mm more apically it had a lower value of 3,196, indicating an age-related decrease in (more apically situated) cementum.

The median density of all abnormal (aplastic/hypoplastic or carious) cementum (*N* = 16 sites) was 501 HU, (range: −692 to 1,343 HU; total range of 2,035 HU). Cementum with a moderate degree of cemental hypoplasia (*n* = 5 sites) anatomically and on microCT imaging including the caudal infundibulum of tooth C, 15 mm subocclusally (Figure 8) that had a HUof 1,132, whilst its rostral infundibulum with more marked hypoplasia had a HU value of 475 (Figure 8).

Cementum with gross hypoplasia/aplasia (*n* = 4 sites) as shown in both infundibulae in **Figure 10** (i.e., Tooth D 30 mm subocclusally) had a HU value of −396 for its rostral infundibulum that contained cementum of moth-eaten appearance with basophilic pockets of mineralization. Its caudal infundibulum containing just stands of collagen-like tissue had an even lower HU value of −560. At a more apical level (image not shown) this caudal infundibular cement had the least dense area recorded (−692 HU).

Infundibular cementum with caries and containing food material, e.g., the rostral infundibulum of tooth A 30 mm subocclusally (Figure 3) had a HU value of 99, whilst at 40 mm subocclusally (Figure 4) this infundibulum had a HU value of 22. Tooth E, 15 mm subocclusally (**Figure 11**) had less severe carious changes in its rostral infundibulum with a HU value of 1,265 HU and unusually, a lower density of 717 in its *caudal* infundibulum. The tooth most severely affected with caries contained just necrotic food material in its infundibulae with no cementum available for measurement (**Figure 10**).

## Discussion

### Micro-CT Findings

Micro-computed tomography (microCT) allowed assessment of cheek tooth anatomy in much greater detail than the conventional CT imaging used to examine 200 infundibulae in a companion study (8), without the physical disruption of dental structure (including complete enamel loss by the prolonged decalcification process) that is necessary for histological examination of these large calcified structures. While a standard CT scan image volumes of 1 mm^3^, a microCT scanner can image sections as small as 1–5 μm^3^ (16). Similar types of infundibular abnormalities were identified using micro-CT and standard CT imaging of in the companion study (8). However, MicroCT images allowed more detailed evaluation of infundibular anatomy and also allowed localized measurement of infundibular tissue attenuation.

Some microCT images showed areas of marked cemental hypointensity that were larger than the lesions observe on gross and histological sections (Figures 4, 8, 9). In these infundibulae, some hypointense cemental areas that appeared to be completely devoid of cementum on MicroCT imaging were histologically found to contain very porous, likely minimally calcified cementum (Figures 4, 8, 9). However, cementum with reduced calcification would not be identified histologically, because all calcified tissues are fully removed during histological preparation. Additionally, the HU values of these hypointense areas also confirmed that they were not completely devoid of tissue, showing that CT can overestimate the size of cemental lesions.

### Histological Findings

Only one of 16 infundibulae (6.25%) examined by MicroCT and grossly had no detectable cemental defects grossly or histologically (in the 7 teeth that could be examined). The remaining 15/16 infundibulae had cemental defects with some examples outlined in Figures 3–**11**. All infundibulae contained an obvious central vascular channel with multiple horizontal branches extending peripherally through the cementum, including into the buccal infoldings in some infundibulae. These buccal infoldings are documented to be the first infundibular areas to be filled with cementum (3, 9) but in this study, they had cemental lesions similar to those observed in more central areas of some infundibulae. The larger central cemental defects were often irregularly shaped (Figures 2–**11**) but the lateral branches were typically cylindrical and tapered in a peripheral direction. These branches varied from empty to containing pale eosinophilic cellular debris, fragmented cemental debris, and/or food material. Some very hypoplastic areas had an irregular, moth-eaten appearance (Figure 9). These findings are similar to those of a recent study (7) where extensive lateral vascular branching surrounding a variably-sized central vascular channel was histologically present in all infundibular cementum sections examined.

Cemental caries was sometimes accompanied by disrupted vascular channel architecture, i.e., with carious destruction and thus irregularity of the usually well-defined cylindrical vascular site walls by caries (Figures 4, 9). Whilst some cemental defects contained some food particles (e.g., Figures 3, 4, 8) it is likely that most intra-infundibular food material was lost during decalcification and histological preparation.

Because all enamel is removed during decalcification of specimens just leaving an empty “enamel space,” the enamel changes seen on microCT and gross sections (e.g., Figures 10, 11) could not be assessed histologically in these specimens. Kilic et al. (1) histologically described abnormal infundibular cemental architecture, similar to the hypoplastic cementum findings in the current study, with up to one-third of cemental areas being composed of tortuous vascular channels, particularly in areas with gross cemental hypoplasia (1).

Some infundibulae had expansion of “normal” sized occlusal defects in an apical direction (as shown between Figures 3, 4 and between Figures 8, 9) a feature also noticed on standard CT imaging in a companion study (8). Despite having relatively “normal” sized occlusal defects, some of these infundibulae had food penetration into the deeper attached cemental defects (e.g., Figure 8). Due to the prolonged mastication of horses (intermittently for up to 18 h/day) with massive masticatory occlusal pressures (of up to 2,000 Newtons) (17) it appears that even the smallest “normal” occlusal defects can have food deeply impacted within them—as also occurs with narrow, valve-shaped cheek teeth diastemata (18). This finding further raises the question of what could be considered normal, in terms of dimensions of a central vascular channel.

The presence of food material, which could serve as a bacterial growth substrate, several centimeters below the occlusal surface, even in teeth with a normal sized central vascular channel defect, could allow significant caries to form prior to occlusal exposure of these deeper hypoplastic cemental areas, and early clinical detection of such subocclusal lesions may not be possible by routine dental examinations, that would just show a “normal” sized central vascular channel.

### Density of Infundibular Cementum

In this study, the HU values for infundibular cementum on microCT imaging were generally higher than the reported HU values obtained by standard CT imaging. Currently, MicroCT imaging can only be performed on extracted teeth and it is also possible that the absence of alveoli and supporting bones may also have influenced infundibular cemental HU values as compared to standard CT imaging in a living horse. Using microCT, tissues of similar attenuation to water, such as soft tissue and fluids, have HU values similar to those obtained by standard (helical) CT. However, tissues with a higher mineral content (and thus with a higher attenuation than water) such as bone or mineralized dental tissues, react very differently when absorbing radiation on microCT than on a standard (helical) CT scan (20), for example, the HU of bone on microCT imaging can reach up to 7,000 HU.

For this reason, the median microCT assessed HU of normal cementum in this study (3,438 HU) was much higher than the value of 1,852 HU reported for equidae cementum using standard CT imaging (9, 19). In this study, infundibular cementum of normal appearance also had a broad HU range on microCT, i.e., from 2,029 to 3,725 HU. Such a broad HU range in bone would indicate that different types of bone (such as cancellous or cortical) were present, with a typical range of 2,000 HU between these two types of bone found on CT (20). The above wide HU range for equine infundibular cementum recorded in this study indicates that the structure of apparently normal cementum differs markedly between individual teeth. Density also differs between different levels of the infundibulae, with cementum becoming less dense more apically, i.e., the cement that is occlusally exposed in later life, but larger studies are needed to verify this finding.

Most infundibulae in this study contained extensive, lateral horizontal branches off the central vascular channel, which varied in number and size, and from being empty; filled with collagenous debris, food material, or degraded cementum; or combinations of these. It would appear that normal appearing cementum with low numbers of small lateral branches (e.g., Figures 1, 6) has the highest density (up to 3,725 HU) whilst cementum with more extensive and larger lateral branching is less dense (as low as 2,029 HU) as was found in deeper normal cementum (55 mm subocclusally) in Figure 5.

Although the central vascular channel was always visible on microCT, the lateral branches were too small to be imaged using this modality. The resolution of the microCT scanner used in the current study is stated to be 82 μm (XTreme CT Specifications, Scanco Medical AG) which is greater than the 40.75 μm median diameter of normal equine infundibular lateral cemental channels (1).

The minimum microCT HU value for hypoplastic/aplastic infundibular cementum in this study was −692 HU, which is relatively close to the designated attenuation of air (−1,000 HU). This can be explained by the recognized presence of some air in these very hypoplastic or aplastic areas. The maximum value for hypoplastic cementum was 1,343 HU, which is much lower than the minimum value obtained for grossly normal infundibular cementum (2,029 HU). When the density of abnormal cementum was compared with histological findings, carious cementum (containing fragments of cement and food material had a median attenuation of 408 HU. As noted the most carious tooth (tooth F) had no remaining infundibular cementum and could not be assessed, but if any cementum were present, it undoubtedly would have had even lower HU values. Overall, the severity of cemental hypoplasia and caries was seen to vary greatly. The limited numbers of observations presented here will represent a limited range of possible cemental densities.

## Conclusion

This study showed that infundibular cemental hypoplastic and/or carious lesions appeared as hypointense lesions on MicroCT imaging with the degree of hypointensity varying with the degree of cemental developmental absence or carious loss. MicroCT appeared to overestimate the size of some lesions as compared to gross and histological examinations, due to the apparent presence of hypo-mineralized cementum in some lesions. Histologically, hypoplastic infundibular areas consisted of an often irregularly-shaped, wide central channels, with multiple, cylindrical side-branches that extended peripherally to a variable extent. Cementum with high numbers of wide empty channels or cementum of more irregular moth-eaten appearance had a low density on CT. Infundibular areas completely devoid of cementum and only containing fragments of collagen-like material and air, were sometimes present more apically and had HU values close to that of gas. Carious areas had connections with the occlusal surface with disruption of their cemental architecture and disrupted central and lateral vascular channels containing food and cemental debris.

## Ethics Statement

This study was approved by the Ethical Review Committee of the Royal (Dick) School of Veterinary Studies and the Roslin Institute, The University of Edinburgh on 12th February 2012.

## Author Contributions

AH contributed to the study design and execution, data analysis and interpretation, and manuscript preparation. SS contributed to study design and execution and interpretation, and manuscript preparation. PD contributed to the study design and execution, data interpretation, and manuscript preparation.

### Conflict of Interest Statement

The authors declare that the research was conducted in the absence of any commercial or financial relationships that could be construed as a potential conflict of interest.
